# Plant factory technology lights up urban horticulture in the post-coronavirus world

**DOI:** 10.1093/hr/uhac018

**Published:** 2022-02-19

**Authors:** Li Zhang, Xiao Yang, Tao Li, Renyou Gan, Zheng Wang, Jie Peng, Jiangtao Hu, Junling Guo, Yang Zhang, Qingming Li, Qichang Yang

**Affiliations:** 1 Institute of Urban Agriculture, Chinese Academy of Agricultural Sciences (IUA-CAAS), Chengdu National Agricultural Science and Technology Center (NASC), Chengdu, 610213, China; 2 Institute of Environment and Sustainable Development in Agriculture, Chinese Academy of Agricultural Sciences, Beijing, 100081, China; 3 BMI Center for Biomass Materials and Nanointerfaces, School of Biomass Science and Engineering, Sichuan University, Chengdu, 610065, China; 4 Key Laboratory of Bio-resource and Eco-environment of Ministry of Education, College of Life Sciences, Sichuan University, Chengdu, 610065, China

Dear Editor,

The pandemic of novel coronavirus disease 2019 (COVID-19) has highlighted the critical importance of ensuring a consistent supply of horticultural products (e.g. vegetables and fruits) [1]. Worldwide quarantine and social distancing led to transportation disruptions, labor shortages, and limited access to local markets, all of which had a significant impact on the production, post-harvest processing, distribution, and consumption of horticultural products in urban areas. Moreover, the traditional agricultural approach is currently facing the unprecedented challenge of feeding an expanding population, as approximately 6.7 billion people are expected to live in urban areas by 2050. Rapid urbanization brings great challenges to horticultural production: gradual shrinking of arable land, declining numbers of agricultural practitioners, reduced availability of irrigation water for farming, increased costs of food transportation, and exacerbation of environmental deterioration. Thus, the supply of horticultural products to urban areas will depend critically on whether such farming systems can enable steady and effective production, a stable and balanced supply, shortened distribution chains, and consistent availability and accessibility of products without compromising safety concerns. In this regard, plant factories with artificial light (PFALs) represent an innovative and promising production system that has shown great potential for stable, effective production of horticultural products both during and after the COVID-19 crisis.

**Figure 1:**
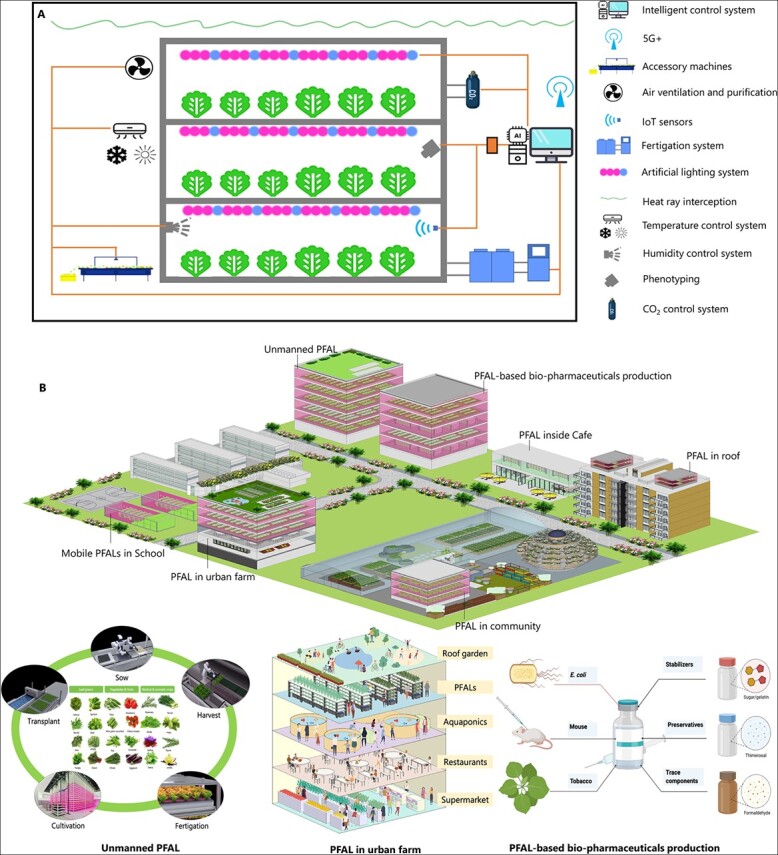
**Characteristics and functions of PFALs. A,** A typical PFAL is composed of several systems, including an external building envelope, a cultivation system, an environmental control system (temperature, humidity, and CO_2_), an air ventilation and purification system, artificial lighting, a fertigation system, an intelligent control system, and accessory machines. PFALs demonstrate the following advantages: i) vertical cultivation technology that increases the productivity of horticultural crops, with an annual lettuce production per m^2^ of 120 kg, more than 40 times that of the open field [2]; ii) high-yield and high-quality products that are free from contamination and contain high concentrations of phytochemicals; iii) closed cultivation systems that maximize the use of internal resources (e.g. water use efficiency >96%, fertilizer use efficiency >80%) [2]; iv) high ability to control the microenvironment around the crops, enabling easy monitoring of plant development; v) continuous production throughout the year; vi) no geographical restrictions, allowing for shorter transportation distances and a smaller carbon footprint during transportation; vii) higher levels of mechanization with reduced labor costs; and viii) a stable food supply regardless of the climate and pandemics. **B,** In the post-coronavirus era, PFALs will serve a variety of purposes in urban agriculture. PFALs have the potential to contribute significantly to the supply of urban horticultural products, the production of high-quality food, and the alternative manufacture of plant-based biopharmaceuticals. They could be associated with urban activities, enriching a plentiful “vegetable basket” and offering individuals a healthy diet.

PFALs are multi-layer indoor farming systems that employ green and sustainable crop cultivation techniques, including vertical cultivation, optimized lighting recipes, energy-conserving technology, and intelligent control systems to enable horticultural production regardless of climatic and geographic conditions (Fig. 1a) [2, 3]. The global indoor farming market reached 32.3 billion USD by 2020, with over 500 PFALs being operated commercially in China, Japan, Singapore, the US, and the UK. Simultaneously, there has been intensive research on increasing the productivity and sustainability of PFALs and on producing high-quality horticultural products in PFALs in recent years.

Vertical cultivation technology significantly increases horticultural productivity through the development of new lightweight materials for structural bracing frames and high-rise modular assembly layers and the application of operating technologies (e.g. operating machinery, auxiliary robots, and automation equipment) [3]. In addition, the enhanced light efficacy and optimization of coupled environmental factors significantly promote plant growth and photosynthesis in PFALs. Numerous investigations have focused on developing “lighting recipes” to optimize lighting conditions (e.g. intensity, spectrum, and photoperiod) for a variety of purposes, including high yields, quality, and energy efficiency, depending on the plant species and the stage of growth and development. Several studies have aimed to apply advanced lighting systems to maximize plant light interception and provide uniform illumination across all leaf surfaces while simultaneously co-optimizing other environmental factors [3, 4].

Improving energy use efficiency to reduce production costs and maximize economic benefits is crucial for the environmentally sustainable development of PFALs. For instance, establishing energetic fluxes and applying a flexible yield-energy model could help researchers understand the energy balance and optimize the control strategy for weather conditions in order to minimize energy consumption [5]. Moreover, it will be important to reduce electricity consumption for lighting and air conditioning by implementing multi-factor refined management, generating electrical energy from clean energy sources (e.g. solar, wind, biomass, and geothermal energy), and storing excess electricity in stationary batteries for time-adjustable usage with higher efficiency.

Rapid development of Internet of Things (IoT) devices—including optical, mechanical, and electrochemical IoT sensors, communication technologies, methods for massive Internet connectivity, data storage, and processing units—has paved the way for IoT applications in PFALs. In addition, artificial intelligence-based data analytics is a powerful tool for automation and decision-making in agriculture, and IoT-enabled accessory machinery can help increase crop productivity and reduce human resource requirements [6]. Future intelligent control systems for PFALs are expected to incorporate multiple platforms with functions such as order management, seed plot scheduling, production management, environmental control, energy consumption measurement, intelligent decision support, and market forecasting, thus enabling the complete control of unmanned PFALs to achieve cost-effectiveness, conserve resources and energy, and reduce labor requirements.

Cultivating nutritious and healthy horticultural products will be an important goal for agricultural development in the future, especially in the post-coronavirus world. Breeding cultivars that are suited to the constraints imposed by PFALs could provide a breakthrough, with the goal of selecting cultivars that are fast growing, high yielding, high quality, functional, and tolerant of low light with a high edible fraction and high light and nutrient use efficiency. For example, Kwon et al. (2020) bred a compact, early-flowering tomato variety suitable for urban agriculture by editing tomato genes for stem length regulation (*SlER*), condensed shoots, rapid flowering (*SP5G*), and precocious growth termination (*SP*) [7].

PFALs serve a variety of functions in urban horticulture, including provisioning (e.g. horticultural produce and biopharmaceutical supply), social activity (e.g. popularization of horticultural knowledge), and cultural activity (e.g. recreation and amenities) (Fig. 1b). Horticultural plants cultivated in PFALs contribute to a plentiful “vegetable basket” and healthy diets for urban dwellers. Because pre-harvest factors such as lighting, temperature, humidity, CO_2_, fertilizers, and fertigation conditions can be precisely adjusted in PFALs, it is possible and feasible to produce horticultural products with multiple objectives, including high concentrations of specific phytochemicals and high-yield products. Vaccines have played a critical role in preventing disease during the COVID-19 pandemic. Currently, PFAL-based manufacturing systems are recognized as a viable alternative source of biopharmaceutical materials and vaccine candidates, and they are being proposed for the automated, standardized, high-yield production of a variety of biopharmaceuticals, including peptide antigens, recombinant vaccines, and virus-like particles [8]. For example, virus-like particle vaccines containing functional hemagglutinin for pandemic influenza can be produced in plants within a month, whereas egg- and cell-based vaccines take 4–6 months to develop. When combined with gene-editing technology, PFALs may enable the development of numerous novel, necessary vaccines. PFALs can also allow people to farm on and inside urban buildings and support the recovery and productive transition of vacant office space. This type of agricultural system is often associated with leisure and recreational functions [9]. PFALs could also be established inside shopping buildings, be equipped with highly modern vertical cultivation technologies, and integrate production of quality horticultural food with services such as workshops, catering, and entertainment events.

In conclusion, PFALs represent rapidly developing and promising horticultural crop cultivation systems that can produce fresh crops in an environmentally friendly manner. PFALs show great potential for addressing the most challenging issues in agricultural science and associated fields, such as population growth, water scarcity, loss of arable land, food safety, and supply chain challenges. They will thus undoubtedly play an important role in the agricultural revolution, food security, carbon neutralization, and the future of humankind. PFALs employ several green and sustainable approaches, thereby representing a shift away from horticultural practices based on human intuition and experience towards urban and modern horticulture based on precise data management. Innovation within PFALs will promote the future integration of current agricultural practices with other rapidly developing techniques (e.g. artificial intelligence, advanced materials, synthetic biology) and help to achieve the global objectives of sustainable agriculture. Thus, PFALs represent a critical solution for urban horticulture in the post-coronavirus world.
